# Specific types of femoral head fractures: be alert for pre-, intra-, and post-operative ipsilateral femoral neck fractures following fracture-dislocation of the femoral head

**DOI:** 10.1186/s40634-023-00666-0

**Published:** 2023-10-13

**Authors:** Shenghui Wu, Jiong Mei

**Affiliations:** Department of Orthopaedic Surgery, Shanghai Sixth People’s Hospital Affiliated to Shanghai Jiao Tong University School of Medicine, Shanghai, China

**Keywords:** Posterior hip dislocation, Femoral head fracture, Femoral neck fracture, Propensity score matching

## Abstract

**Purpose:**

Ipsilateral femoral head and neck fractures (iFHNFs) are rare types of fractures that confer extremely poor prognosis among femoral head fractures (FHFs). Owing to the rarity of FHFs, it is challenging to diagnose iFHNFs. In addition, the clinical features of iFHNF have not yet been comprehensively elucidated. Therefore, this retrospective study aimed to summarize and analyze the clinical characteristics of iFHNF using a clinical diagnostic simulation based on a prospectively maintained database.

**Methods:**

Clinical data of consecutive patients with FHFs, including gender, age, injury side, and associated injuries, were collected and analyzed from a prospectively maintained orthopedic database at a large level-I trauma center for a clinical diagnostic simulation. Patients were stratified according to the presence or absence of iFHNF. Moreover, propensity score matching (PSM) was used to create 1:1 age- and gender-matched couples. Lastly, clinical factors were compared and identified between the two groups before and after matching.

**Results:**

A total of 218 FHF patients were included. Fifteen patients were diagnosed with ipsilateral femoral neck fractures (iFNFs), including preoperative, intraoperative, and postoperative types. There were 177 male and 41 female patients, with a mean age of 40.0 ± 16.5 years. The incidence of two factors, namely acetabular fracture and posterior hip dislocation, were significantly different between the two groups (*P* < *0.05*). Following PSM, 15 pairs of patients were generated. Comparisons revealed that the incidence of posterior hip dislocation was significantly different between the two groups (*P* < *0.05*).

**Conclusions:**

There were three types of iFHNFs. In the context of FHFs, posterior hip dislocation was associated with iFNFs. Thus, surgeons should remain vigilant, not only intraoperatively but also postoperatively, for iFNFs following FHF and concomitant posterior hip dislocation.

**Level of Evidence:**

Diagnostic level IV

**Supplementary Information:**

The online version contains supplementary material available at 10.1186/s40634-023-00666-0.

## Introduction

Femoral head fractures (FHFs) are relatively rare injuries usually caused by high-energy trauma. They typically occur after a posterior dislocation, and approximately 5% to 15% of posterior hip dislocations involve FHFs [1]. FHFs may also occur simultaneously with acetabular fractures, with an estimated incidence of 29.2% [2]. In addition, the incidence of ipsilateral femoral head and neck fractures (iFHNF) is low [3]. However, iFHNFs, as a special type of fracture, have the worst prognosis among all FHFs [3–5]. Attributed to the complex anatomy of the hip joint, it is difficult to restore the natural structure of the hip joint to gain complete recovery of function following iFHNF [3]. In addition, the clinical manifestations of iFHNF have not yet been fully elucidated. Hence, a comprehensive understanding of iFHNF may assist in guiding the management of FHFs.

This study aimed to summarize the characteristics of iFHNFs and other types of FHFs to identify their differences using a clinical diagnostic simulation based on a prospectively maintained database. We hypothesize that there are common characteristics between iFHNFs and other types of FHFs.

## Methods

### Subjects

This retrospective study was conducted in accordance with the Declaration of Helsinki (2013 revision). The study was approved by the Human Research Ethics Committee of our institution before initiation [No. 2022-KY-026(K)], and individual consent was waived by the Human Research Ethics Committee owing to the retrospective nature of the study.

To perform the clinical diagnostic simulation, a prospectively maintained orthopedic database at a large level-I trauma center was used. A retrospective search was conducted for the Computed Tomography (CT) imaging data of patients diagnosed with FHF between January 1, 2011, and August 1, 2022. Then, patients with FHFs were consecutively enrolled. Three investigators independently reviewed the imaging data of all FHFs to radiographically confirm the diagnosis. Exclusion criteria included poor-quality CT images (i.e., images with severe artifacts), unclosed epiphysis, pathological fracture, or skeletal immaturity.

Clinical data, including gender, age, injury side, associated anterior hip dislocation, associated central hip dislocation, associated posterior hip dislocation, and associated acetabular fracture, were collected. Analyses of associated injuries were discussed to reach a consensus by two fellowship-trained trauma surgeons for all cases.

### Statistical analysis

Statistical analyses were performed with R version 3.5.3. Qualitative data were presented as numbers (percentages), whereas quantitative data were expressed as mean (SD). Patients were stratified according to the presence or absence of iFHNF. Differences between groups were compared using the chi-square test or Fisher exact probability method for dichotomized values. Continuous variables following normal distribution were compared using the Student t-test. Data were analyzed with the nonparametric Mann–Whitney U test if normality or variance tests failed. To minimize the effects of potential confounding factors, Propensity Score Matching analysis (PSM) was applied for age and gender. Matching (1:1) on the propensity score was performed using a nearest neighbor-matching algorithm with a caliper of 0.02 of the propensity score. After PSM, differences between the two groups were re-assessed. *P* < 0.05 was considered statistically significant.

## Results

### Patient characteristics

**Fig. 1:**
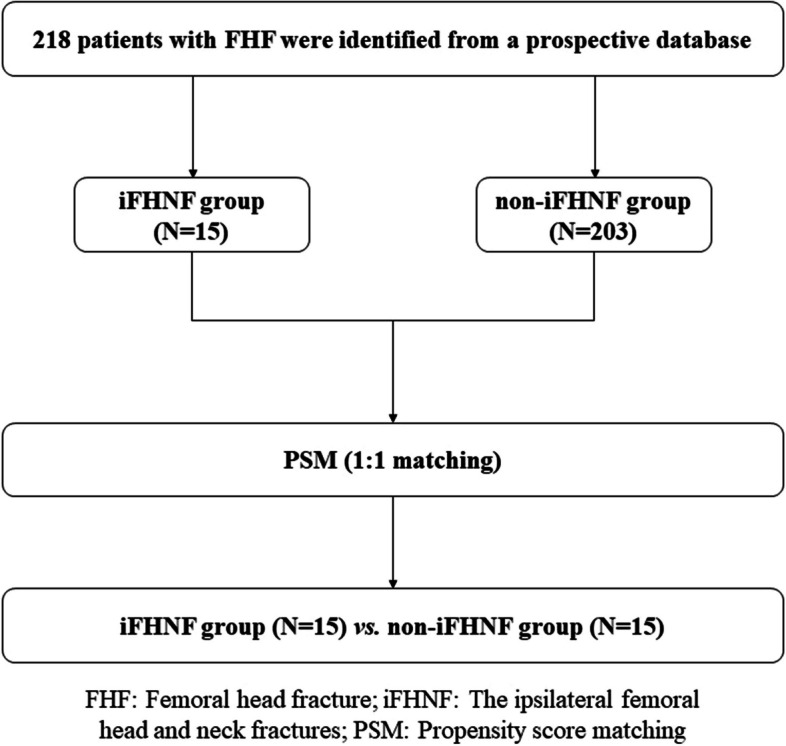
Study flow chart

**Fig. 2:**
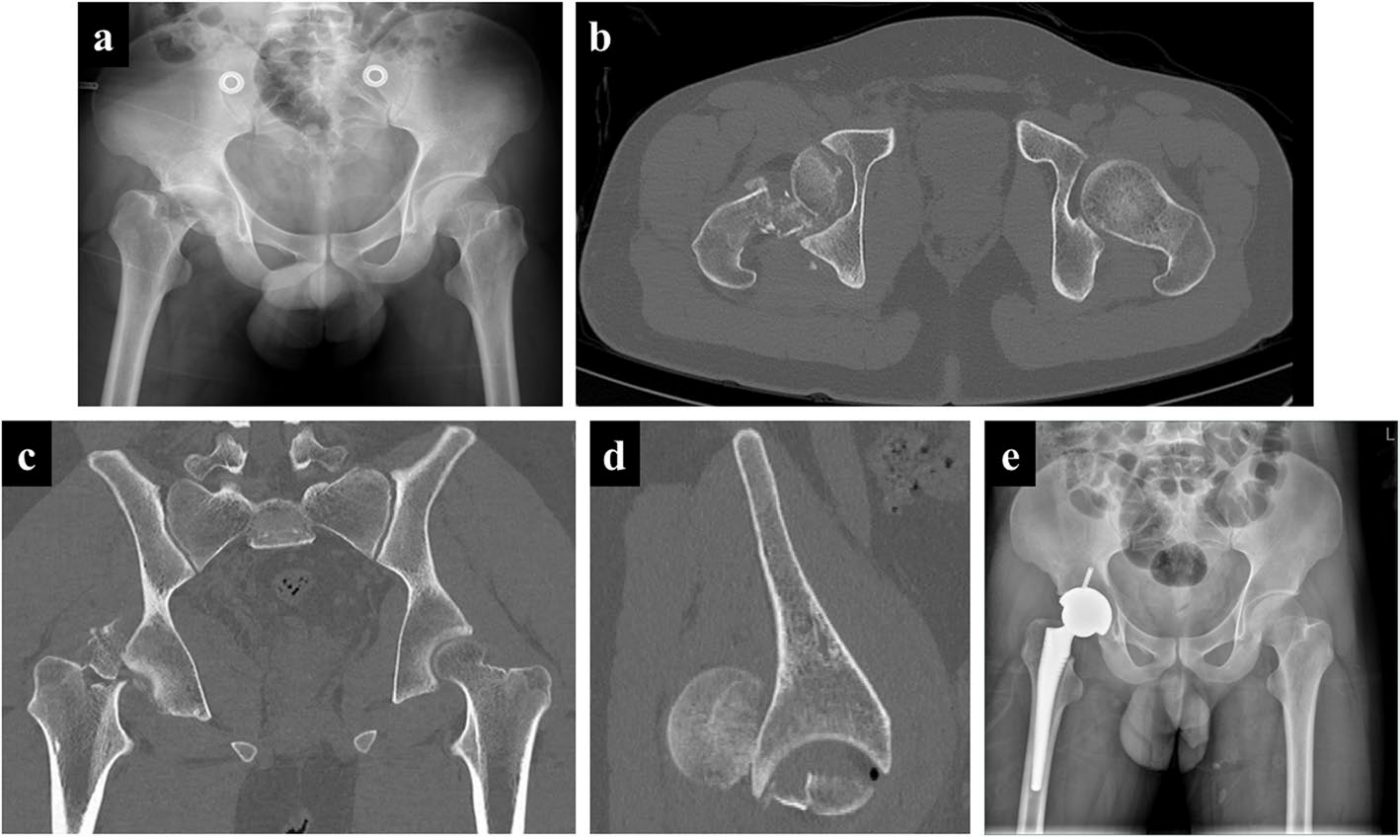
A representative case of the first subtype of iFHNF, namely posterior dislocation and fracture of the femoral head with simultaneous femoral neck fracture. A 24-year-old man involved in a severe traffic accident was diagnosed with femoral head and neck fractures based on X-ray (**a**) and CT images (**b**-**d**). A large extra-articular fragment of the femoral head and posterior hip dislocation can be observed (**d**). Total hip arthroplasty was performed (**e**)

**Fig. 3:**
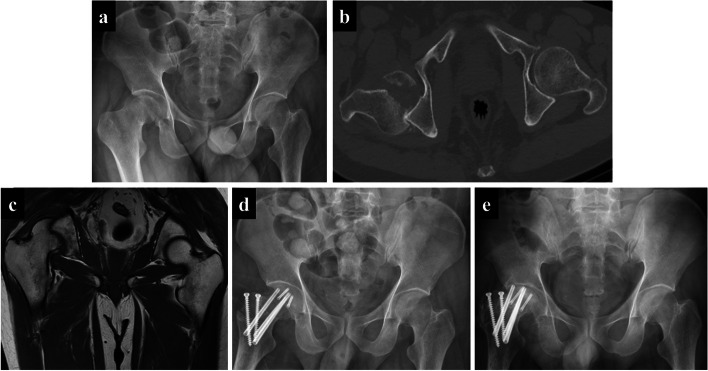
A representative case of the second subtype of iFHNF, namely posterior dislocation and fracture of the femoral head with subsequent femoral neck fracture. A 42-year-old man involved in a severe traffic accident was diagnosed with femoral head fracture and posterior hip dislocation based on X-ray (**a**) and CT images (**b**). No visible indications of bone fractures were observed in the femoral neck on MRI images (**c**). However, the femoral neck was fractured during open reduction and internal fixation. Thus, the femoral head and neck were internally fixed (**d**). Finally, femoral head necrosis was detected (**e**)

**Fig. 4:**
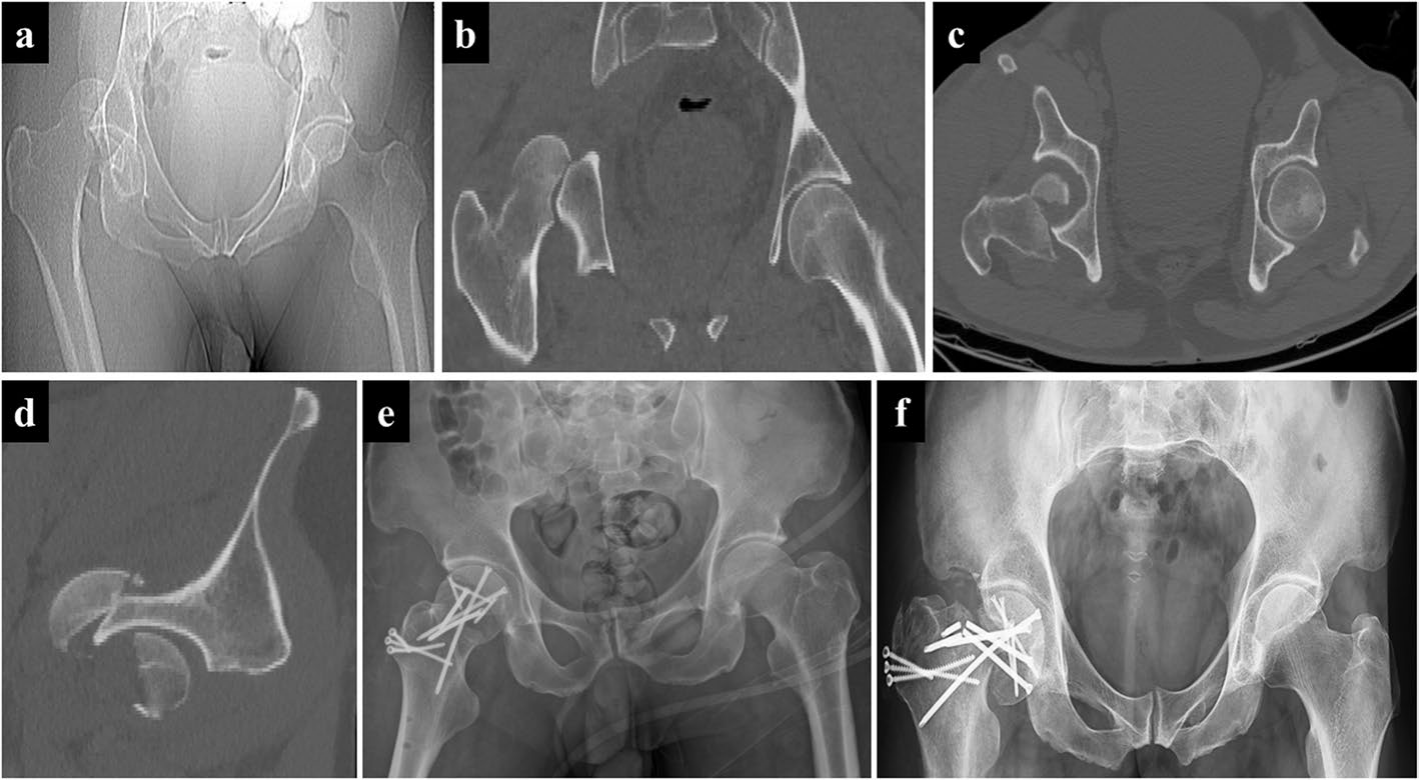
A representative case of the third subtype of the iFHNF, namely posterior dislocation and fracture of the femoral head with postoperative femoral neck fracture. A 43-year-old man involved in a severe traffic accident was diagnosed with femoral head fracture and posterior hip dislocation based on X-ray (**a**) and CT images (**b**-**d**). Open reduction and internal fixation of the femoral head fracture was conducted (**e**). Finally, the femoral neck refractured without trauma (**f**)

A total of 218 patients with FHFs were eligible to participate in this retrospective study, including 15 patients with iFHNF. The study flow chart is illustrated in Fig. 1. Conjointly, there were three trauma types of iFHNF (Figs. 2, 3 and 4). Ten patients were diagnosed preoperatively, with one patient suffering from refracture of the femoral neck during CT examination. Two patients were intraoperatively diagnosed upon close reduction. Moreover, three patients suffering from femoral neck fractures were diagnosed postoperatively. Patient characteristics and features of FHFs are summarized in Table 1. There were 177 male and 41 female patients, with a mean age of 40.0 ± 16.5 years. The most frequent associated injury was acetabular fracture occurring in 142 (65.1%) patients, whilst the most frequent associated hip dislocation was posterior dislocation, occurring in 126 (57.8%) patients.

**Table 1:** Demographics and radiographic characteristics of patients with femoral head fractures

Characteristics	Before PSM	After PSM
Total (no. [%])	218(100.0%)	30(100.0%)
FHF with FNF (no. [%])	15(6.9%)	15(50.0%)
Side of injury (no. [%])		
Left	107(49.1%)	15 (50.0%)
Right	111(50.9%)	15 (50.0%)
Age (yr)	40.00 ± 16.52	39.87 ± 16.69
Sex (no. [%])		
Male	177(81.2%)	25(83.3%)
Female	41(18.8%)	5(16.7%)
Associated injuries (no. [%])		
Acetabulum fracture	142(65.1%)	14(46.7%)
Anterior dislocation	5(2.3%)	0(0)
Central dislocation	10(4.6%)	2(6.7%)
Posterior dislocation	126(57.8%)	19(63.3%)

*PSM* Propensity Score Matching, *FHF* Femoral Head Fracture, *FNF* Femoral Neck Fracture

### Clinical data analysis

Clinical factors were compared between the iFHNF group and the non-iFHNF group. Interestingly, the incidence of two factors, namely acetabular fracture and posterior hip dislocation, were significantly different between the two groups (*P* < 0.05) (Table 2). After PSM, 15 pairs of patients were generated. The clinical characteristics of the 30 patients are listed in Table 1. There were 14 (46.7%) patients with acetabular fractures and 19 (63.3%) with posterior hip dislocation. In addition, the incidence of posterior hip dislocation was significantly different between the two paired groups (*P* < 0.05) (Table 2).

**Table 2:** Comparisons of the difference in the clinical factors between two groups classified by the ipsilateral femoral head and neck fractures

Variable	Before PSM			After PSM		
	iFHNF	non-iFHNF	*p*-value	iFHNF	non-iFHNF	*p*-value
Number	15 (100)	203 (100)		15 (100)	15 (100)	
Side of injury			0.7329*			1.0000^†^
Left	8 (53.3)	99 (48.8)		8 (53.3)	7 (46.7)	
Right	7 (46.7)	104 (51.2)		7 (46.7)	8 (53.3)	
Age	41.6 ± 20.81	38.0 (26.0–50.0)	0.9341^#^	41.6 ± 20.81	38.13 ± 11.73	0.7242^#^
Sex			1.0000^†^			1.0000^†^
Male	12 (80.0)	165 (81.3)		12 (80.0)	13 (86.7)	
Female	3 (20.0)	38 (18.7)		3 (20.0)	2 (13.3)	
Acetabulum fracture	5 (16.7)	137 (67.5)	0.0074^*^	5 (16.7)	9 (60.0)	0.2723^†^
Anterior dislocation	0 (0)	5(2.5)	1.0000^†^	0 (0)	0 (0)	-
Central dislocation	0 (0)	10(4.9)	1.0000^†^	0 (0)	2 (13.3)	0.48284^†^
Posterior dislocation	15 (100)	111 (54.7)	0.0006^*^	15 (100)	4 (26.7)	< 0.0001^†^

*PSM* Propensity Score Matching, *iFHNF* The ipsilateral femoral head and neck fractures

^*^Number of patients (percentage) and *p*-values determined with the chi-square test

^#^Median (interquartile range) and *p*-values derived with the Mann–Whitney U test

^†^Number of patients (percentage) and *p*-values determined with the Fisher exact test

### Characteristics of iFHNF

In this study, iFHNFs, as a special injury type, included but were not limited to Type III Pipkin fracture or the intraoperative type. Totally there were three subtypes of iFHNFs. The classical cases manifested as posterior hip dislocation combined with one of the three types of iFHNF. More specifically, the iFHNF types consisted of preoperative ipsilateral femoral head fracture, femoral neck fracture (Fig. 2), intraoperative femoral neck fracture (Fig. 3), and postoperative femoral neck fracture (Fig. 4). Concerning postoperative fractures, three patients presented femoral neck fracture at one, three, and seven months after surgery, respectively.

## Discussion

The results validated our hypothesis that there were common characteristics in patients with iFHNFs. There were three subtypes of iFHNFs, including preoperative, intraoperative, and postoperative ones. Prior to PSM, two factors, namely acetabular fracture and posterior hip dislocation, were associated with iFHNF. However, posterior hip dislocation was only associated with iFHNF after PSM. It is worthwhile emphasizing that the high incidence of acetabular fracture masked the role of posterior hip dislocation in iFHNF. To the best of our knowledge, this is the largest consecutive case series of FHFs summarizing the characteristics of iFHNFs. To date, iFHNF has the most abysmal prognosis among all types of FHFs [3–5]. Due to the rarity of FHFs, standard FHF management guidelines have not yet been established. Hence, the characteristics of this particular type of FHF could be used to develop an accurate fracture model, thereby clarifying the mechanism of injury, guiding treatment, and finally improving the prognosis of these patients.

### Comparison with literature data

A PubMed search, limited to English-language literature, was performed using the search terms "femoral head fracture" AND "femoral neck fracture." This search screened twelve titles (Supplemental Table S1). Abstracts were judged for relevance. In case of doubt, the full articles were read, and cross-references were checked (Supplemental Figure S1). Finally, 17 clinical studies on the iFHNF, ipsilateral femoral head fracture with femoral neck fracture were selected (Supplemental Table S2). The incidence of iFHNF with posterior hip dislocation, revealed herein to be significantly different between the two groups, was compared with literature data extracted from all selected studies. Studies on the iFHNF are shown in Table 3. Likewise, the number of iFHNF and posterior hip dislocation was extracted in all eligible studies. Unexpectedly, the incidence of posterior hip dislocation in iFHNF was 100%, based on previous literature reports. Similarly, it was also 100% in the current study. According to earlier studies, iFHNFcan be categorized into two types, namely Pipkin III [6–9] or iatrogenic Pipkin III [4, 5, 10, 11]. Although this triad was described by Alyousif et al. [10] in a case report, its criteria were not clearly defined.

**Table 3:** Comparison with available literature data on the ipsilateral femoral head and neck fractures

Study (Year)	Study design	Cases (No.)	Country	Time period	Cases of the iFHNF (No.)	Cases of the PHD (No.)	Frequency (%)	Ref
Hougaard et al. (1988)	Cohort	201	Denmark	1958–1985	1	1	100	[6]
Marchetti et al. (1996)	Cohort	38	USA	1979–1993	2	2	100	[8]
Mehta et al. (2008)	Cohort	72	USA	2000–2006	1	1	100	[12]
Ross et al. (2012)	Review	1	USA	2012	1	1	100	[13]
Kokubo et al. (2013)	Case series	12	Japan	1991–2009	2	2	100	[7]
Lawrence et al. (2013)	Case report	1	USA	2013	1	1	100	[14]
Jangir et al. (2014)	Case report	1	India	2014	1	1	100	[15]
Park et al. (2016)	Case series	9	Korea	2010–2015	5	5	100	[5]
Snoap et al. (2016)	Case report	1	USA	2016	1	1	100	[16]
Zhao et al. (2017)	Case report	1	China	2017	1	1	100	[9]
Keong et al. (2019)	Case report	1	Singapore	2019	1	1	100	[4]
Pascarella et al. (2019)	Cohort	69	Italy	2002–2016	2	2	100	[17]
Alyousif et al. (2021)	Case report	1	Saudi Arabia	2021	1	1	100	[10]
Li et al. (2022)	Case report	1	China	2022	1	1	100	[11]
This work (2022)	Cross-sectional study	218	China	2011–2022	15	15	100	-

*iFHNF* The ipsilateral femoral head and neck fractures, *PHD* Posterior hip dislocation

### High incidence of acetabular fracture

Attributed to the high incidence of acetabular fracture, an in-depth examination is essential to avoid misdiagnosis during the management of femoral head fractures. According to prior studies, the incidence of femoral head fracture with acetabular fracture can be as high as 3 times that of femoral head fracture with femoral neck fracture [3, 18]. As anticipated, the incidence of the femoral head fracture associated with acetabular fracture was high in this study. Although acetabular fracture was not associated with iFHNF after PSM, the incidence of acetabular fractures was still non-negligible. In addition, acetabular fractures are one of the most common severe injuries. Consequently, a thorough examination should be carried out to identify acetabular fractures following the diagnosis of FHFs. Moreover, our results demonstrated that iFHNF was only associated with posterior hip dislocation after PSM. Thus, the elements of these complex associated injuries were revealed, including femoral head fracture, femoral neck fracture, and posterior hip dislocation.

### Clinical characteristics of iFHNFs

Posterior hip dislocation can play a decisive role during iFHNF. Previous studies described a potential relationship between posterior hip dislocation and femoral neck fracture in patients with FHFs [5, 19], in line with our results, which echoed the mechanism of this injury. After posterior hip dislocation, the force can lever the dislocated head against the iliac wing, triggering femoral neck fracture [20]. What's more, initial posterior hip dislocation can also be observed concurrently with the femoral neck fracture. The incidence of posterior hip dislocation in iFHNF in the present study was consistent with those reported in previous studies (Table 3). Hence, a deeper understanding of this special injury model could enhance awareness in the clinical setting, given that posterior hip dislocation is common but frequently ignored.

Generally, iFHNF is a rare occurrence commonly caused by high-energy trauma and may be potentially overlooked due to the subtle radiographical findings or the presence of other distracting complex injuries. Moreover, there was an association between the femoral neck fracture and femoral head fracture with posterior hip dislocation both in the timing and the mechanism of injury. The time interval between femoral neck fracture and femoral head fracture with posterior hip dislocation is variable (Figs. 2, 3 and 4). As a result of posterior hip dislocation, the fracture site of the femoral head is attached to the posterior acetabular wall, thus leading to high stress at the femoral neck. Subsequently, micro-structural lesions caused by high stress induce femoral neck fracture. Our findings exposed that femoral neck fracture and posterior hip dislocation can simultaneously occur (Fig. 2) either during treatment or after treatment (Figs. 3 and 4). Therefore, there are three subtypes of iFHNF that require additional attention.

Regarding posterior hip dislocation, femoral head fracture accompanied by femoral neck fracture is the most frequent fracture type of iFHNF. Moreover, this injury type, clinically termed type Pipkin 3 based on the Pipkin classification, is associated with an unfavorable prognosis, whereas the prognosis of subtypes of non-Pipkin 3 fractures remains controversial [3–5, 21]. Eventually, hip replacement surgery is required for type III Pipkin fractures. In addition, type 3Pipkin fracture was also reportedly associated with posterior hip dislocation [6–9]. Consequently, such type of iFHNF did not seem to be amenable to conventional therapy for FHFs or FNFs and needs to be further explored separately.

The second type of iFHNF, namely femoral head fracture with intraoperative femoral neck fracture, is referred to as iatrogenic femoral neck fracture and commonly occurs during closed fracture reduction. The fracture type turned from the non-iFHNF to the iFHNF. Hence, the prognosis of the second type of iFHNF is comparable to that of the first one. In the past few decades, the second type of iFHNF has garnered extensive attention. There were some similar reports on the presence of the second type of iFHNF during the treatment in the literature [4, 5, 10, 11]. Nevertheless, such injuries could not be clinically identified in a timely and effective manner because no potent ancillary diagnostic tools were available. Hence, surgeons must suspect the presence of the second type of iFHNF during the closed reduction procedure. Based on this trauma model, more clinical and biomechanical studies could be carried out.

The rarest type of iFHNF, the third type, is a femoral head fracture with posterior hip dislocation, followed by postoperative femoral neck fracture. To the best of our knowledge, this type has not yet been reported in studies. This fracture type may be similar to the second type of iFHNF. Due to the complexity of this severe injury, iFHNF can develop at any time during treatment. In addition, impairments to the femoral neck may be easily missed, so the third type of iFHNF can be difficult to recognize clinically. Consequently, these occult injuries urge surgeons to prioritize the femoral head and neglect the femoral neck during internal fixation. The biomechanical strength of the femoral neck was likely too low in intensity to meet the stability of bone structures surrounding the fracture site, resulting in postoperative non-traumatic femoral neck fracture. Hence, following FHF with posterior hip dislocation, surgeons should also remain vigilant for the occurrence of femoral neck fracture after surgery. Furthermore, risk factors related to the third type of iFHNF require further exploration.

In patients with occult femoral neck fractures, "prophylactic" fixation of the femoral neck (or the injured lower limb) can be considered in the case of femoral head fracture with posterior hip dislocation. FHFs are generally caused by high-energy trauma, and these patients suffer from significant bone and joint injury. Our findings uncovered that posterior hip dislocations may act as a "trigger" for occult femoral neck fractures. One patient suffered from the preoperative type of iFHNF during CT examination. Its presence was associated with the postural adjustments of the lower limb deformity caused by the posterior hip dislocation. So, injured limb immobilization and special care, such as soft pads for maintaining patients’ position, are recommended during the examination. In addition, some intraoperative femoral neck fractures may occur in an attempt to reduce posterior hip dislocation in femoral head fractures [4, 5, 10, 11], and this may be an intraoperative complication or post high-energy traumatic sequelae. This study also noted that some occult femoral neck fractures may only become apparent post-operatively. Also, there was no radiographic evidence of osteonecrosis of the femoral head in this study. It is difficult to determine if these fractures were caused by the initial trauma or if they were insufficiency fractures caused by bone loss or necrosis. Therefore, "prophylactic" fixation of the femoral neck could be performed in cases of femoral head fracture with posterior hip dislocation to prevent intraoperative and postoperative refractures. It may consequently be a way of reducing femoral neck fracture in the management of FHFs since it can not lead to an increase in operative time and difficulty of surgery. Further research is warranted to investigate the mechanisms of injury and feasible preventive strategies.

Great care would be taken to avoid iatrogenic injury that may cause pre-, intra-, and post-operative ipsilateral femoral neck fractures following fracture-dislocation of the femoral head. The activation of the hip external rotator muscles would lead to an augmentation of the compressive forces exerted on the femoral head, consequently causing modifications in the loading patterns of the hip joint [22]. Therefore, it is recommended that patients diagnosed with fracture-dislocation of the femoral head refrain from engaging in active hip external rotation. Additionally, it is advised to minimize passive hip external rotation, both during preoperative examination and surgical maneuvers.

### Limitations

This study had some limitations. First, the relatively small sample size did not satisfy the requirements for an Event Per Variable. Consequently, binary logistic regression analyses were not performed. However, owing to the rarity of the iFHNF, the results had clinical interpretability. In addition, in contrast with previous studies, the sample size was larger for this type of fracture, which represented a strength of this study. Moreover, posterior hip dislocation has also been associated with iFHNF in clinical reports (Table 3). Secondly, several common clinical factors in FHFs were analyzed in this clinical study, and other factors, including biomechanical, molecular, genetic factors, and other comorbidities, could be identified in further biomechanical studies and clinic-related research.

## Conclusions

In summary, our study identified three types of the iFHNF (ipsilateral femoral head fracture with femoral neck fracture). The postoperative type of iFHNF may be occult. Notably, posterior hip dislocation was closely related to iFHNF. The high incidence of acetabular fracture masked the role of posterior hip dislocation in iFHNF. The concept of iFHNF and posterior hip dislocation was used to distinguish it from other types of femoral head fractures. Emphasis should be placed on iFHNF, which has an extremely poor prognosis. Thus, surgeons should remain vigilant for the presence of femoral neck fractures during not only the intraoperative period but also the postoperative period, especially in cases of femoral head fracture with posterior hip dislocation. Furthermore, the three injury models of iFHNF and posterior hip dislocation can also provide a theoretical basis for future clinical and biomechanical studies to investigate and pioneer optimal treatment approaches. 

### Supplementary Information

**Additional file 1: Figure S1.** Flow chart of selection for relevant articles on the ipsilateral femoral head and neck fractures. **Table S1.** Details of searched literature data in English. **Table S2.** Details of selected literature data and cross-references.

## Data Availability

The datasets used and/or analysed during the current study are available from the corresponding author on reasonable request.

## Abbreviations

iFHNF: Ipsilateral femoral head and neck fracture
FHF: Femoral head fracture
PSM: Propensity score matching
