# Supplementary material for: Specific types of femoral head fractures: be alert for pre-, intra-, and post-operative ipsilateral femoral neck fractures following fracture-dislocation of the femoral head
Source: J Exp Orthop. 2023 Oct 13;10:104. doi: 10.1186/s40634-023-00666-0 (PMC10570253; doi:10.1186/s40634-023-00666-0)
Supplement: Supplementary file 1 — Additional file 1: Figure S1. Flow chart of selection for relevant articles on the ipsilateral femoral head and neck fractures. Table S1. Details of searched literature data in English. Table S2. Details of selected literature data and cross-references. [file 40634_2023_666_MOESM1_ESM.docx]

**Supplementary Materials**

Supplementary Figure

**
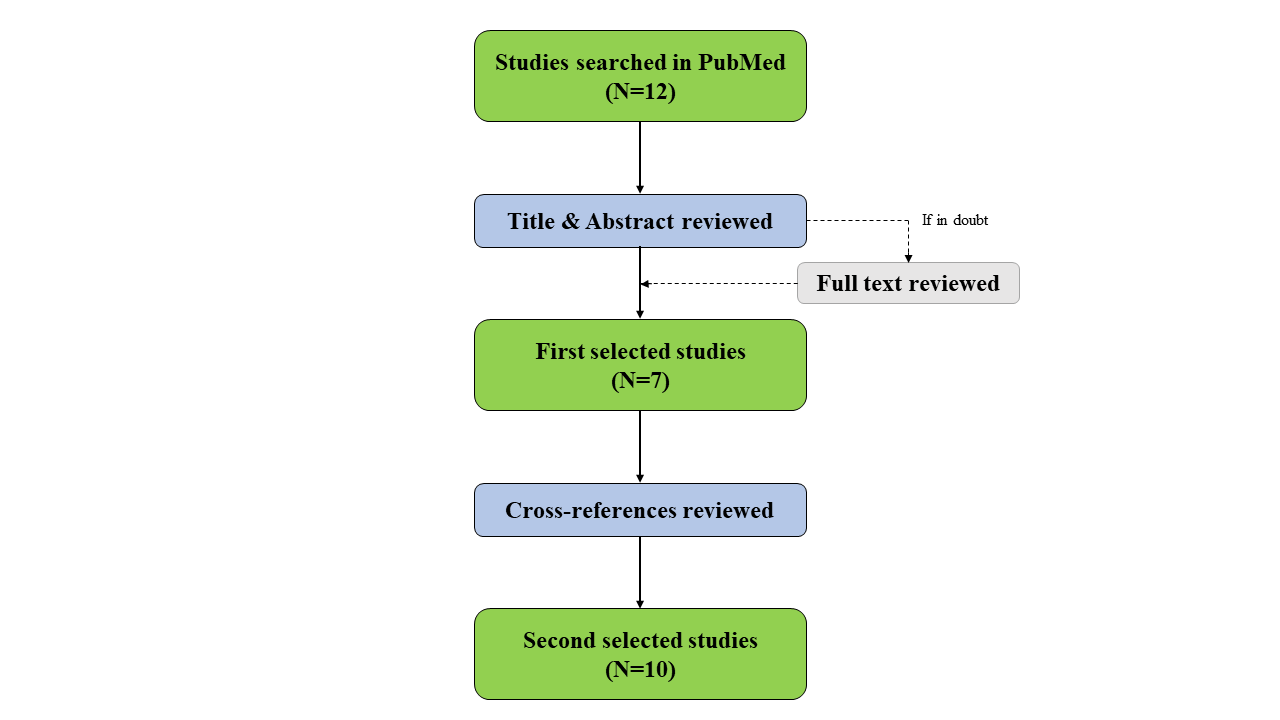
**

Figure S1 Flow chart of selection for relevant articles on the ipsilateral femoral head and neck fractures.

Supplementary Tables

Table S1 Details of searched literature data in English

| No. | Article title |
| --- | --- |
| 1 | Grigsby PW, Roberts HL, Perez CA. Femoral neck fracture following groin irradiation. Int J Radiat Oncol Biol Phys. 1995 Apr 30;32(1):63-7. doi: 10.1016/0360-3016(95)00546-B. PMID: 7721641. |
| 2 | Krawczyk A, Płochowski J, Dragan S, Orzechowski W, Kulej M, Koprowski P. Elliptical head hemiarthroplasty in the treatment of femoral neck and head fractures--preliminary report. Ortop Traumatol Rehabil. 2008 Jul-Aug;10(4):331-7. English, Polish. PMID: 18779766. |
| 3 | Clegg TE, Roberts CS, Greene JW, Prather BA. Hip dislocations--epidemiology, treatment, and outcomes. Injury. 2010 Apr;41(4):329-34. doi: 10.1016/j.injury.2009.08.007. Epub 2009 Sep 30. PMID: 19796765. |
| 4 | Tonetti J, Ruatti S, Lafontan V, Loubignac F, Chiron P, Sari-Ali H, Bonnevialle P. Is femoral head fracture-dislocation management improvable: A retrospective study in 110 cases. Orthop Traumatol Surg Res. 2010 Oct;96(6):623-31. doi: 10.1016/j.otsr.2010.03.020. Epub 2010 Aug 21. PMID: 20729157. |
| 5 | Lombardi AV Jr, Cameron HU, Della Valle CJ, Jones RE, Paprosky WG, Ranawat CS. What would you do?: challenges in hip surgery. J Bone Joint Surg Br. 2012 Nov;94(11 Suppl A):70-4. doi: 10.1302/0301-620X.94B11.30510. PMID: 23118386. |
| 6 | Jangir R, Mishra D. An Unusual Variant of Pipkin's Fracture Dislocation of Hip: A Case Report. J Orthop Case Rep. 2014 Jul-Sep;4(3):19-21. doi: 10.13107/jocr.2250-0685.187. PMID: 27298974; PMCID: PMC4719317. |
| 7 | Park KH, Kim JW, Oh CW, Kim JW, Oh JK, Kyung HS. A treatment strategy to avoid iatrogenic Pipkin type III femoral head fracture-dislocations. Arch Orthop Trauma Surg. 2016 Aug;136(8):1107-13. doi: 10.1007/s00402-016-2481-1. Epub 2016 Jun 6. PMID: 27271755. |
| 8 | Keong MW, Razak HRBA, Koon WM, Ping CC. Case Report of a Rare Pipkin Type III Femoral Head Fracture. J Orthop Case Rep. 2019;9(5):11-15. doi: 10.13107/jocr.2019.v09.i05.1510. PMID: 32547994; PMCID: PMC7276619. |
| 9 | Pascarella R, Fantasia R, Sangiovanni P, Maresca A, Massetti D, Politano R, Cerbasi S. Traumatic hip fracture-dislocation: A middle-term follow up study and a proposal of new classification system of hip joint associated injury. Injury. 2019 Aug;50 Suppl 4:S11-S20. doi: 10.1016/j.injury.2019.01.011. Epub 2019 Jan 17. PMID: 30683569. |
| 10 | Rana R, Verma D Jr, Behera S 2nd, Behera H, Raulo B. Irreducible Femur Head Fracture-Dislocation Treatment With Kocher-Langenbeck Approach With Flip Trochanteric Osteotomy: A Novel Approach. Cureus. 2020 Dec 8;12(12):e11969. doi: 10.7759/cureus.11969. PMID: 33425543; PMCID: PMC7788007. |
| 11 | Alyousif H, Aleisawi H, Alkaff H, Albusayes N. Terrible triad of the hip: A case report. Int J Surg Case Rep. 2021 May;82:105758. doi: 10.1016/j.ijscr.2021.105758. Epub 2021 Mar 14. PMID: 33773958; PMCID: PMC8178459. |
| 12 | Li QW, Zhou CS, Li YP. Case report of a delayed iatrogenic Pipkin type III femoral head fracture-dislocation. Medicine (Baltimore). 2022 Jan 28;101(4):e28773. doi: 10.1097/MD.0000000000028773. PMID: 35089256; PMCID: PMC8797484. |

Table S2 Details of selected literature data and cross-references

| No. | Article title | Cited by Ref. |
| --- | --- | --- |
| 1 | Jangir R, Mishra D. An Unusual Variant of Pipkin's Fracture Dislocation of Hip: A Case Report. J Orthop Case Rep. 2014 Jul-Sep;4(3):19-21. doi: 10.13107/jocr.2250-0685.187. PMID: 27298974; PMCID: PMC4719317. | _ |
| 2 | Park KH, Kim JW, Oh CW, Kim JW, Oh JK, Kyung HS. A treatment strategy to avoid iatrogenic Pipkin type III femoral head fracture-dislocations. Arch Orthop Trauma Surg. 2016 Aug;136(8):1107-13. doi: 10.1007/s00402-016-2481-1. Epub 2016 Jun 6. PMID: 27271755. | _ |
| 3 | Keong MW, Razak HRBA, Koon WM, Ping CC. Case Report of a Rare Pipkin Type III Femoral Head Fracture. J Orthop Case Rep. 2019;9(5):11-15. doi: 10.13107/jocr.2019.v09.i05.1510. PMID: 32547994; PMCID: PMC7276619. | _ |
| 4 | Marchetti ME, Steinberg GG, Coumas JM. Intermediate-term experience of Pipkin fracture-dislocations of the hip. J Orthop Trauma. 1996;10(7):455-61. doi: 10.1097/00005131-199610000-00002. PMID: 8892144. | 3 |
| 5 | Pascarella R, Fantasia R, Sangiovanni P, Maresca A, Massetti D, Politano R, Cerbasi S. Traumatic hip fracture-dislocation: A middle-term follow up study and a proposal of new classification system of hip joint associated injury. Injury. 2019 Aug;50 Suppl 4:S11-S20. doi: 10.1016/j.injury.2019.01.011. Epub 2019 Jan 17. PMID: 30683569. | _ |
| 6 | Kokubo Y, Uchida K, Takeno K, Yayama T, Miyazaki T, Negoro K, Nakajima H, Sugita D, Takeura N, Yoshida A, Baba H. Dislocated intra-articular femoral head fracture associated with fracture-dislocation of the hip and acetabulum: report of 12 cases and technical notes on surgical intervention. Eur J Orthop Surg Traumatol. 2013 Jul;23(5):557-64. doi: 10.1007/s00590-012-1027-7. Epub 2012 Jul 5. PMID: 23412160. | 5 |
| 7 | Scolaro JA, Marecek G, Firoozabadi R, Krieg JC, Routt MLC. Management and radiographic outcomes of femoral head fractures. J Orthop Traumatol. 2017 Sep;18(3):235-241. doi: 10.1007/s10195-017-0445-z. Epub 2017 Feb 10. PMID: 28188487; PMCID: PMC5585088. | 5 |
| 8 | Rana R, Verma D Jr, Behera S 2nd, Behera H, Raulo B. Irreducible Femur Head Fracture-Dislocation Treatment With Kocher-Langenbeck Approach With Flip Trochanteric Osteotomy: A Novel Approach. Cureus. 2020 Dec 8;12(12):e11969. doi: 10.7759/cureus.11969. PMID: 33425543; PMCID: PMC7788007. | _ |
| 9 | Hougaard K, Thomsen PB. Traumatic posterior fracture-dislocation of the hip with fracture of the femoral head or neck, or both. J Bone Joint Surg Am. 1988 Feb;70(2):233-9. PMID: 3343268. | 8 |
| 10 | Ross JR, Gardner MJ. Femoral head fractures. Curr Rev Musculoskelet Med. 2012 Sep;5(3):199-205. doi: 10.1007/s12178-012-9129-8. PMID: 22628176; PMCID: PMC3535084. | 8 |
| 11 | Alyousif H, Aleisawi H, Alkaff H, Albusayes N. Terrible triad of the hip: A case report. Int J Surg Case Rep. 2021 May;82:105758. doi: 10.1016/j.ijscr.2021.105758. Epub 2021 Mar 14. PMID: 33773958; PMCID: PMC8178459. | _ |
| 12 | Mehta S, Routt ML Jr. Irreducible fracture-dislocations of the femoral head without posterior wall acetabular fractures. J Orthop Trauma. 2008 Nov-Dec;22(10):686-92. doi: 10.1097/BOT.0b013e31818e2a86. PMID: 18978543. | 11 |
| 13 | Lawrence DA, Smitaman E, Baumgartner M, Haims A. A rare but radiographically recognizable cause of an irreducible hip fracture-dislocation. Clin Imaging. 2013 May-Jun;37(3):595-7. doi: 10.1016/j.clinimag.2012.06.009. Epub 2012 Aug 13. PMID: 23601776. | 11 |
| 14 | Snoap T, Freyder J, Roberts J. Management of a Combined Femoral Head and Neck Fracture: A Case Report. JBJS Case Connect. 2016 Oct-Dec;6(4):e88. doi: 10.2106/JBJS.CC.16.00066. PMID: 29252742. | 11 |
| 15 | Zhao B, Li H, Yan J, Han LR, Yang XF. Pipkin type III femoral head fracture-dislocation combined with complicated acetabular fracture: A rare case report and literature review. Medicine (Baltimore). 2017 Dec;96(50):e9214. doi: 10.1097/MD.0000000000009214. PMID: 29390346; PMCID: PMC5815758. | 11 |
| 16 | Yu X, Pang QJ, Chen XJ. Clinical results of femoral head fracture-dislocation treated according to the Pipkin classification. Pak J Med Sci. 2017 May-Jun;33(3):650-653. doi: 10.12669/pjms.333.12633. PMID: 28811788; PMCID: PMC5510120. | 11 |
| 17 | Li QW, Zhou CS, Li YP. Case report of a delayed iatrogenic Pipkin type III femoral head fracture-dislocation. Medicine (Baltimore). 2022 Jan 28;101(4):e28773. doi: 10.1097/MD.0000000000028773. PMID: 35089256; PMCID: PMC8797484. | _ |
